# Exploring Australian Health Promotion Practitioners’ Awareness, Understanding and Application of Theories of the Policy Process in Health Promotion Practice: A Qualitative Study

**DOI:** 10.34172/hpp.45504

**Published:** 2026-06-06

**Authors:** Rhian Jones, Kara Lilly, Rachel Cole

**Affiliations:** School of Health, University of the Sunshine Coast, Maroochydore, Australia

**Keywords:** Health promotion, Health policy, Policy making, Public policy

## Abstract

**Introduction:**

Theories of the policy process provide structured ways to understand, navigate and influence political landscapes using evidence-based approaches. While influencing policy is a core function of the health promotion discipline, little is known about how practitioners engage with these theories. This study aims to address this gap by examining health promotion practitioners’ awareness, understanding, and application of theories of the policy process in practice, and factors influencing health promotion practitioners’ engagement in policy processes.

**Methods:**

A thematic qualitative study was used to explore perspectives and experiences of health promotion practitioners practicing in Queensland, Australia using online, semi-structured interviews. Data were analysed with NVivo software using a reflexive thematic analysis to identify key themes.

**Results:**

Four key themes were generated; 1) health promotion practitioners understand policy process complexity but have limited awareness of theories of the policy process, 2) health promotion practitioners implicitly apply constructs of theories of the policy process in their practice, 3) organisational context shapes the extent to which health promotion practitioners’ are positioned to engage in policy processes, and 4) industry-level factors influence health promotion practitioners’ engagement in policy processes.

**Conclusion:**

Opportunities exist for health promotion practitioners to more explicitly draw from theories of the policy process to inform their practice in influencing healthy public policy for population health and equity outcomes. Stronger inclusion of theories of the policy process within tertiary education and professional development, including formalising industry accreditation, may support strengthening and maintaining practitioner skills to meet industry core competencies.

## Introduction

 Contemporary health promotion recognises that a person’s ability to control their health depends on various interconnected factors influenced by their situation and surroundings, known as the determinants of health.^1,2^ According to the World Health Organization, these determinants may directly or indirectly account for 30-55% of health and wellness outcomes.^3^ One structural way health promotion practitioners address these determinants is through healthy public policies; the deliberate actions or decisions made by governments that regulate or guide aspects of human life and influence population health outcomes.^4^ However, addressing contemporary complex public health challenges, or ‘wicked’ problems of the 21^st^ century using healthy public policy, requires comprehensive policy processes and cooperation across all sectors,^5^ including those outside of the healthcare system.^6^ Successful examples of healthy public policy have included tobacco control measures, such as restrictions on smoking in public spaces,^7^ and motor vehicle safety regulations, such as mandatory seatbelt use.^8^ The policy development and assessment skills required to undertake healthy public policy-making have been recognised as essential competencies for health promotion practitioners since the 1980s.^5,9,10^ Today, these skills are embedded in health promotion competency frameworks in the United States,^11^ Europe^12^ and Australia.^13^

 Healthy public policymaking involves the complex interaction of stakeholders, ideologies and contextual factors, reflecting the multifaceted and dynamic system that shapes population health outcomes.^9,14^ Traditionally, health promotion practitioners have drawn on linear theories of the policy process, such as the stages heuristic framework,^15^ which suggest a logical progression through agenda setting, policy formation, policy implementation, and evaluation. However, critics argue that these theories fail to capture the complexities of policy dynamics.^9^ In contemporary contexts, health promotion practitioners have turned to some of the more tried and tested theories of political science pertinent to healthy public policy development – hereafter referred to collectively as theories of the policy process – to better understand these complexities.^9,16^ Each theory provides more unique insights into the complexities of how policies are created, agendas are set, and decisions are made in the policy environment, by identifying theoretical constructs to explain policy-making processes.^6^ For example, the Advocacy Coalition Framework (ACF), one example of a policy process theory, posits that coalitions of like-minded actors achieve policy objectives by collaborating to influence policy outcomes.^17^ These coalitions, driven by shared beliefs and goals, can strategically shape policy agendas and decision-making processes by collectively mobilising resources and reframing perspectives to shift policy direction.^18^ In contrast, the Multiple Streams Theory (MST)^19^ explains how issues, policies, and political contexts intersect to shape policy outcomes across three ‘streams’ – the problem stream, policy stream, and political stream. A window of opportunity for policy change is created when these streams converge.^19^ Furthermore, the Punctuated Equilibrium Framework (PEF) characterises policy change as occurring between long periods of stability (or equilibrium) and moments of punctuated disruption.^20^ During these times of disruption, changes in how issues and policy solutions are framed and decision-making jurisdictions become key points of debate, creating opportunities for health promotion practitioners to advocate for major policy change.^20^ Consistent with the foundational use of theory in health promotion practice,^21^ understanding and employing these dynamic theories of the policy process may support health promotion practitioners to be better able to recognise and leverage policy process opportunities and build legitimacy for successful healthy public policy action.^22^

 International literature using theories of the policy process for understanding healthy public policy development and adoption in a research context is growing.^17,23,24^ For example, a recent study exploring physical activity policy development in French municipalities utilised theories of the policy process to conceptually model the contextual influences on policy development across dynamic policy lifecycles.^25^ Other emerging research investigating policy processes in obesity prevention highlighted the value of theoretical conceptualisation of public policy processes for understanding the drivers of healthy public policy adoption in the Australian context.^17,26-28^ Despite growth in this policy process research, research on the application of theories of the policy process in health promotion practice in the field remains sparse. Historically, evidence has suggested that health promotion practitioners are inadequately equipped with the skills needed to effectively influence healthy public policy processes,^10^ attributed to ‘naivety’ on theories of the policy process.^9^ In more recent years, studies have found that health promotion students and graduates report limited understanding of legislative processes,^23^ with policy processes often drawing on simplified models that fail to reflect the complexity of real-world policymaking.^22^ This narrowed understanding and application of theories of the policy process suggests that health promotion practitioners may remain ill-equipped with the strategic acumen necessary to navigate the complex and often unpredictable environments of healthy public policymaking.^29^ This study aims to investigate if this assumption is true, by exploring health promotion practitioners’ awareness, understanding and application of theories of the policy process, based on their professional experience in practice. Additionally, this study seeks to explore factors influencing health promotion practitioners’ engagement in policy processes in practice.

## Methods

###  Study design

 An exploratory qualitative study was conducted using semi-structured online interviews underpinned by constructivist epistemology. Two experienced health promotion academics with industry experience (KL and RC), and one university student in the process of completing a health promotion-related undergraduate honours research degree (RJ) made up the research team. This brought valuable insider knowledge but also necessitated ongoing reflection to recognise and mitigate potential personal and professional biases. The dual role of the researchers as both academics and experienced industry practitioners in the field informed the design of the interview guide and shaped the interpretation of participant experiences through a reflexive lens. Throughout the interviews, the researchers maintained a reflexive stance by taking field notes and engaging in post-interview debriefs to reflect on interviewer influence, potential assumptions, and the co-construction of meaning in the interview setting. Ethical approval was granted by the University of the Sunshine Coast Human Research Ethics Committee (S231868).

###  Sampling

 Criterion-based purposive sampling^30^ was used to identify potential participants within the research team’s professional networks, allowing participants to be selected based on their knowledge and experience of policy processes to ensure data were relevant and informative.^31^ Eligible participants included practitioners in Queensland, Australia who self-identified as being currently employed in a position with predominant role functions and responsibilities aligned with the Core Competencies for Health Promotion Practitioners.^13^ Additionally, all participants self-identified as having a qualification in health promotion (inclusive of Bachelor’s Degree, Graduate Certificate, Graduate Diploma, Master’s Degree or PhD).

 Twenty-three eligible participants were approached to participate in an interview via email containing a Research Project Information Sheet and consent form for participants to sign and return to indicate their interest in the study. A snowball sampling technique was applied to identify further eligible participants, encouraging invited participants to forward the invitation email to their professional networks or recommend other practitioners for involvement in the study. Consenting participants were emailed to organise a convenient interview time using Google Calendar, along with the interview questions to review and prepare for the interview.

###  Data collection

 All interviews were conducted by two members of the research team via each participant’s preferred online meeting platform (e.g., Zoom^32^). Interviews were conducted between January and March 2024, taking 30 to 60 minutes each. A semi-structured interview guide (see Supplementary file 1) was used to facilitate the discussions and allow flexibility to pursue issues raised by participants. Consistent with a reflexive qualitative approach, the interview questions were informed by reflexivity underlying action^33^ critical reflection constructs, and were structured to explore the experiences, perspectives, and practical applications of theories of the policy process of health promotion practitioners in health promotion practice. Reflexivity also informed the design and conduct of interviews by prompting the research team to consider how their own professional backgrounds in health promotion and policy might shape question framing, follow-up prompts, and interpretations during the interviews and data collection. Questions aligned to critical reflection constructs were designed to encourage participants to critically appraise their practice and interpret the various influences that shape their approach to navigating the policy process (Table 1).

**Table 1:** Survey question development matrix

**Research focus**	**Question focus**	**Reflexivity underlying action construct/s**	**Question/s**
Awareness	Awareness of theories of the policy process	Knowledge validity	Q5. Can you describe any policy process theories or frameworks that can be applied to health promotion practices?
Understanding	Understanding of constructs of theories of the policy process	Knowledge validity	Q5. Can you describe any policy process theories or frameworks that can be applied to health promotion practices? Q6. Can you describe an example of how you have applied a specific policy process theory in your health promotion work? If yes, please specify the theory or theories.
	Understanding of influences on policy processes in practice	Underlying values, beliefs and assumptions Influences shaping practice Implicit power dynamics	Q4. In your experience, what factors influence policy decisions in health promotion practice?
Application	Use of theories of the policy process in practice	Knowledge validity	Q6. Can you describe an example of how you have applied a specific policy process theory in your health promotion work? If yes, please specify the theory or theories.
	Effectiveness and usefulness of applying theories of the policy process in practice	Knowledge validity Underlying values, beliefs and assumptions Implicit power dynamics	Q6 (a). (IF answered Question 6 with a specific example) *How was the use of policy process theory useful for guiding health promotion actions in your work?*
	Barriers/challenges to implementing policy processes in practice	Underlying values, beliefs and assumptions Influences shaping practice Implicit power dynamics	Q4. In your experience, what factors influence policy decisions in health promotion practice? Q7. In your opinion, what are some potential challenges or barriers to navigating policy processes in real-world health promotion initiatives?
	Enablers for engaging in policy processes in practice	Knowledge validity Influences in shaping practice Implicit power dynamics	Q4. In your experience, what factors influence policy decisions in health promotion practice? Q8. What additional resources or support do you think may be useful for health promotion practitioners to effectively engage in healthy public policy processes?

 During interviews, interviewers adopted a reflexive stance by remaining attentive to how their assumptions, positioning, and interview style might influence participant responses, and by using open-ended probing questions to privilege participants’ meanings rather than confirm preconceived ideas. Interviews were recorded and converted to MP3 format, prior to being professionally transcribed. A member of the research team (RJ) reviewed the transcripts, removing any identifying information to ensure anonymity. Each participant was provided with a copy of their transcript for review, with the opportunity to provide additional information, request changes or remove content. Each participant was assigned a pseudonym name, which was used throughout the analysis and reporting stages of the study to maintain confidentiality.

###  Data analysis

 Interview transcripts were imported into NVivo version 14^34^ for thematic analysis. Following the six-stage reflexive thematic analysis process described by Braun and Clarke,^35^ a systematic approach was used to generate, analyse and report themes within the qualitative dataset. The first stage of coding applied an inductive data-driven approach free from prior assumptions to examine patterns across the data. Further immersion in the data was undertaken to refine the central organising themes. Whilst the authors’ theoretical understanding of the constructs of the ACF, MST and PEF informed interpretive generation of the final codes during this second stage, the purpose of this study was not to systematically map participant accounts and experiences to theoretical frameworks. Rather, theoretical references were used to contextualise and interpret patterns observed in practitioner experiences, consistent with the exploratory aims of the study. Themes were reviewed in relation to their coded extracts to ensure that they were consistent with the data and transcripts were revisited to ensure that all information and viewpoints were included. During analysis, reflexive team discussions were used to challenge interpretations, consider alternative readings of the data, and examine how analytic decisions were being shaped by the researchers’ assumptions and positionality. Transcripts were revisited to ensure that diverse perspectives were represented and that themes were grounded in participants’ accounts rather than driven by theoretical expectations. Themes were finalised through an iterative process of reviewing coded extracts and the full data set, with ongoing reflexive discussions among the research team to refine thematic boundaries, resolve interpretive differences, and ensure themes were coherent, internally consistent and meaningfully represented the dataset as a whole. One researcher (RJ) completed each stage of coding, theming and reviewing, and these were discussed iteratively with the remaining members of the study team (KL and RC) to align with the reflexive approach. This iterative process involved revisiting and refining the steps throughout the analysis, and collaborative reflexive research team engagement supported critical interrogation of interpretations and strengthened the transparency of data analysis.

## Results

 A total of 10 participants took part in the study, with eight recruited through purposive sampling and two via snowball sampling. The majority (n = 6) of participants held an undergraduate degree in health promotion or public health (including Honours) as their highest level of qualification, while the remaining participants (n = 4) had completed a relevant postgraduate degree. Participants had a median of 18 years of experience in the health promotion industry, with a range of one to 24 years. Most participants were in operational or middle management roles (n = 9) and employed by the state government (n = 8).

 Four overarching themes were generated during data analysis; 1) health promotion practitioners understand policy process complexity but have limited awareness of theories of the policy process, 2) health promotion practitioners implicitly apply constructs of theories of the policy process in their practice, 3) organisational context shapes the extent to which health promotion practitioners’ are positioned to engage in policy processes, and 4) industry-level factors influence health promotion practitioners’ engagement in policy processes. Detailed descriptions of the themes are presented below, with illustrative quotes from participants.

###  Health promotion practitioners understand policy process complexity but have limited awareness of theories of the policy process

 Influencing policy for health outcomes was acknowledged by participants as being complex, with policy processes described as convoluted and often requiring years, or even decades, to achieve meaningful reform. One participant described the dynamic and non-linear nature of policy development and implementation as a journey, involving the need to navigate:

 “*…What are the hooks and levers to get what you need through the system? It’s sort of this sideways, up, fun and games that you have to go through to get greater good for the community.”* (Jordan)

 Despite recognising the complex nature of policy processes, participants described experiencing persistent frustrations when attempting to undertake policy practice. Reece explained that this was because they had not been exposed to policy constructs as part of their education or professional development which caused confusion about what the idea of ‘policy’ means in practice. This uncertain conceptual grounding was echoed in the context of undertaking policy processes, with Jamie reflecting that they had learned about theoretical foundations of policy processes during their tertiary education but recalled predominantly using behaviour change theories in practice. Similarly, most participants were unable to recall any theories that could help inform policy practice, with several noting that they were not aware of theories to support and inform policy processes. Participants were more likely to take an intuitive approach, with Charlie explaining that they,

 “*…Usually just throw it all back under supportive environments and health public policy. So, I tend to just use good old Ottawa and go…we’ll change our policy by changing the way we go there.”*

 There was one exception to this, with Chris reporting that they “fell upon” Kingdon’s MST and felt it aligned with shifting policy action:

 “*That’s probably the one I adhere to and use most…I’m not 100 percent sure when I came across it and how I came across it. I remember when I came across it, it immediately resonated with me, and I did some further personal investigation of it.”*

 Mixed responses were reported on the role education played in preparing the participants for undertaking policy practice when they entered the workforce. Several reported feeling prepared and that the education they received equipped them for policy practice, whilst others reported feeling underprepared due to limited understanding of political contexts. Most commonly, participants reported that their understanding of policy processes developed through workplace exposure, particularly by gaining practical experience and achieving tangible policy outcomes in real-world settings. This emphasis on learning through practice was reinforced by participant views that successful policy development was largely in the remit of experienced health promotion practitioners rather than new graduates:

 “*I don’t think you would be a graduate that would step into that sort of role. That’s a much higher role...So, it’s not entry level, it’s very much you would have experience in that…You come in and you do your grassroots work, and you get exposed to it in different ways. Then eventually, if that’s where you want to end up, you end up there.”* (Jordan)

###  Health promotion practitioners implicitly apply constructs of theories of the policy process in their practice

 Although most participants did not identify specific theories of the policy process, they reflected on ways they navigated policy process complexity in practice. Participants described the value of recognising and acting on reform opportunities when they arise, understanding the significance of policy windows that often open and close unexpectedly, and remaining attentive to emerging opportunities in the policy environment. Proactive policy planning was reported as supporting participants in being prepared for these opportunities when:

 “*All of a sudden the policy windows open and you go, oh here is my back pocket document that I did five years ago [laughs]. Have this. All of a sudden, life is just so much easier.”* (Jordan)

 Participants also reported navigating complex policy landscapes by fostering shared visions for policy practice to create a collective platform for advocacy, with the involvement of multiple stakeholders being seen to strengthen influence. The importance of relationship building was seen to be supported by the often-prolonged nature of healthy public policy processes, as Sam explained:

 “*It helps a lot if you actually get to know these people and become almost friends as opposed to advocates. That can make a real difference.” *

 In additional to fostering strong relationships, inter- and intra-industry collaborations were also reported as key to successful policy development and implementation. For instance, Sam described adopting a networked governance approach to build partnerships across organisations that have traditionally operated in isolation, aiming to develop a system-wide policy solution that better addressed the needs of lower-resourced communities. Chris also noted that much of their policy practice focused on recognising the interconnected nature of the factors that influence health outcomes, and

 “*Acknowledging that when we’re trying to implement health promotion policy, we are talking about cross-sector policy, not just health policy…Working with other sectors – predominantly within government…To talk to them and try and influence the highest level – their policies, their strategies.”*

 This capacity to influence policy decision-makers was seen to depend on strategic language use and narrative framing, in particular, the ability to deliver a concise and audience-tailored value proposition. As Jamie explained, they use strategic language to help align political priorities with healthy public policy action by

 “*Making them realise that the people that are voting for them are going to care about these issues…Showing that if they do this, it’s going to make them look good.”*

 Aligning political priorities and political will was viewed as challenging, with participants reporting they often had to engage in negotiations with decision-makers and key policy players to persuade them of the importance of the policy issue being addressed. Participants reported having to present multiple options and create narratives that were sometimes simplified or less contentious to contend with political interests and agendas. This was seen as counterintuitive to their innate reliance on evidence and structured planning practices to guide decisions for policy action. To gain traction, participants explained that they had learned to balance these evidence-based arguments with strategic messaging through the use of storytelling:

 “*Because you can never underestimate the impact of the people that it affects and the impact that it has around the room…It actually helps put a human and a face to what you’re talking about.”* (Alex)

 The timing of messaging and alignment of policy processes with election years was reported as being in the front-of-mind for many experienced participants, with policy processes strategically paced to coincide with key policy windows. These approaches were seen as a way to ensure proposals are both timely and positioned to capture the decision-makers at the most opportune moment. Participants explained that the goal is to have a policy initiative proactively prepared that aligns with an election commitment and can be readily endorsed by decision-makers.

###  Organisational context shapes the extent to which health promotion practitioners are positioned to engage in policy processes

 Participants highlighted several organisational factors that affected their ability to influence policy processes. Competing interests for monetary allocation, budget constraints and funding availability were identified as barriers to engaging in healthy public policy processes. Dedicated financial resourcing for policy action was reported as limited, fragmented and often difficult to access. This impacted participants’ ability to prioritise policy initiatives, with proposals first requiring exploration of whether funding could be allocated. Alex described the implications of this on engaging in public policy processes, stating,

 “*This is a huge barrier for us, because we don’t even get an opportunity to put things forward when there is this ‘no money’ narrative being thrown at us.”*

 Financial constraints were viewed as creating difficult trade-offs when prioritising policy action - particularly when strong evidence supported preventive policy approaches, yet decisions around healthy public policy were guided by cost-effectiveness and the pursuit of maximum return on investment. Several participants reported a key challenge in obtaining funding and support for initiatives was the limited understanding of contemporary health promotion among the general public and organisational leadership. Many participants believed that health promotion continues to be associated with outdated notions of one-way information delivery, such as educational brochures and superficial materials, rather than being recognised as systems- and evidence-based practice. Participants suggested that a better recognition of health promotion practice as a professionally qualified discipline and the contribution that practitioners can make to shaping healthy public policy processes would help to build a broader understanding of the value of contemporary health promotion. Participants reported this as a long-standing challenge that continues to be problematic for health promotion practitioners, with Charlie highlighting that,

 “*The challenges that we faced are the same ones that we had 20 years ago, which is a lack of understanding of what health promotion is. It isn’t me standing in a shopping centre.”*

 Alongside this misconstrued understanding of health promotion, several participants felt that high workforce turnover and limited capacity for undertaking policy action hindered health promotion practitioners’ ability to effectively influence policy processes. Participants reported that frequent staff changes were disruptive to policy process continuity and there was often insufficient capacity to fully implement healthy public policy processes:

 “*That’s part of the problem – you get a group of people…and then they go, and you can’t go and start again with the new people that come in.”* (Chris)

 Supportive leadership was seen to mitigate these obstacles, by creating support and space for meaningful policy action. Participants felt that effective leaders encouraged open communication, critical thinking and professional growth and facilitated opportunities for practitioners to engage effectively with policymakers.

###  Industry-level factors influence health promotion practitioners’ engagement in policy processes

 Participants identified several influencing factors within the health promotion industry that affected their ability to engage in policy processes. They described barriers such as bureaucratic constraints, the nature of political cycles and restructures, and the diminishing role of health promotion functions within state departments. This highlighted the broader issue of limited recognition for the health promotion profession. Participants emphasised the importance of being firmly situated as qualified health practitioners to safeguard their professional identity and ensure their specialised qualifications and expertise are acknowledged. As Alex reflected,

 “*I think standing really strong that we belong in the HP [health practitioner] stream is a big thing…we are health practitioners and we will stay in this stream and be recognised within this stream.”*

 Participants highlighted that professional accreditation could support this recognition of health promotion practitioners as qualified health practitioners with unique and valuable skills. Formalised accreditation was perceived as a means of establishing a recognised standard of professionalism and competence in the field. The accreditation process was described as offering opportunities for professional reflection, demonstrating practitioners’ achievements and competence, and providing greater scope for ongoing professional development. Accreditation was also perceived as a mechanism to strengthen the credibility of practitioners in policy discussions and to reinforce health promotion as a legitimate profession:

 “*It’s the rationale for why that role was important, and that you’ve got this specific training and you’ve done this amount of continued learning or CPD [Continuing Professional Development].”* (Morgan)

 Building on this, several participants suggested that there were unrealised opportunities in fostering stronger collaboration between academia and policy practice. Specifically, the opportunity to broaden the reach of health promotion by embedding relevant content across other discipline curricula was seen as a way to enhance the position of health promotion practice in policy arenas. For health promotion practitioners, this would

 “*…make their life a whole lot easier because then they’re talking to people in other sectors who actually have a basic understanding of health promotion, a connection with their work, with their industry, with their sector…they become advocates and champions within other sectors. To me, that would be where the greatest power lies.”* (Chris)

 Participants also suggested involving experienced practitioners with policy process experience in education to share their practical insights and help students develop a clearer understanding of how policy processes operate in practice to better prepare them for real-world policy environments.

## Discussion

 This study is one of few to examine health promotion practitioners’ awareness, understanding and application of theories of the policy process, as well as the factors influencing their engagement in policy processes. Overall, health promotion practitioners in this study demonstrated a well-developed appreciation of the complexity, dynamism and political nature of policy processes, reflecting many constructs within established theories of the policy process. Few practitioners, however, explicitly identified or consciously applied specific theoretical frameworks to guide their practice. Instead, they relied predominately on experiential knowledge, professional judgement and contextual insights; consistent with the small but growing body of evidence examining practitioner engagement in policy practice.^36^ Similarly, a global scoping review of national-level physical activity and sedentary behaviour policies found that only 15% explicitly applied a policy process framework, further reinforcing the limited use of formal theory in health-related policy development.^37^

 Although health promotion practitioners rarely used formal theories explicitly, many accounts aligned with constructs within established frameworks. Participants described deliberate coalition-building efforts, developing alliances and fostering consensus to amplify collective voices in policy processes, strategies consistent strategies consistent,^18^ which emphasises coalition building among groups with shared beliefs to drive policy change within complex and dynamic political environments. They also acknowledged the inherent instability of these alliances, echoing the ACF’s focus on the challenges of maintaining coalitions over time in fluctuating political settings.^18^ Participants also recognised and acted on ‘policy windows’ described in the MST,^19^ emphasising the need for continual readiness to capitalise on moments when problem, policy and political systems converge. Furthermore, efforts to engage political decision-makers, aligning health issues with political interests, and use of political pressure to build momentum were consistent with the PEF,^20^ which recognises that policy processes can remain stable over time but can experience sudden changes resulting from shifts in public attention, political will, or societal pressures. Practitioners’ use of public pressure and political image to build political support for policy processes reflects the PEF’s focus on leveraging political motivations to catalyse change,^20^ demonstrating an understanding of policy environment (in)stability and the capacity to promote policy shifts when conditions are politically favourable. While these examples demonstrate parallels between health promotion practitioners’ actions and theoretical constructs, examining how practitioners draw on theory-informed reasoning was beyond the scope of this study and remains unclear. Rather than systematically mapping practice to specific theories, the theoretical framing was used to contextualise practitioners’ experiences. Future research could build on these findings by exploring the mechanisms through which practitioners draw on theory-informed reasoning in practice and how more intentional theoretical grounding might strengthen policy engagement. Developing and empirically testing conceptual models that connect practitioner experiences, organisational conditions, and policy process theories may further support theoretical integration in health promotion practice.

 Participants’ reflections also illustrated their function as policy entrepreneurs,^19^ actors who strategically build coalitions, mobilise resources and leverage opportunities to advance healthy public policy - activities closely mirroring core health promotion roles in advocacy, partnership development, and system-level change. A Swedish case study^22^ further reinforces the central role of health promotion practitioners as policy entrepreneurs, demonstrating how applying theories of the policy process can support navigation of dynamic policy environments and enhance understanding of how policy change unfolds in health promotion contexts. Studies such as these illustrate that theories of the policy process provide a valuable lens through which practitioners can interpret and navigate the complexities of policy work.^38^ However, increasing industry awareness of these theories and their practical relevance may be necessary,^39^ including strengthening understanding the policy entrepreneur role for the health promotion sector in supporting the translation of theories of the policy process into locally meaningful practices.^38,39^ This study also highlights a potentially important role for the health promotion sector in supporting the translation of policy theory into practice through on the ground experience, mentoring and effective leadership. Further research could explore how lessons from other sectors that more explicitly integrate policy process theories might inform their application and practical integration within health promotion practice.

 The application of theories of the policy process was also influenced by practitioners’ limited formal preparation for policy work. Many participants reported minimal exposure to the political nature of health promotion during their tertiary education and felt underprepared to navigate policy processes upon entering the workforce. Several described ‘learning on the job’, noting that policy engagement is a skill developed and consolidated over time through practical experience. This may reflect the continued sector-wide focus on traditional programmatic activities that include discrete and easily measurable interventions, rather than investments broader policy-focused or system-oriented approaches that address the environmental and structural determinants of health equity.^40^ In this regard, workplace expectations for health promotion practitioners to engage in healthy public policy would need to be matched with adequate investment in policy-related workforce development and organisational support.

 Although this is the first study to our knowledge to investigate health promotion practitioners’ perceived preparedness for policy engagement upon workforce entry, its findings align with other recent policy-related research. A US study^23^ found that while 78% of health promotion students viewed public policy action as beneficial, most lacked understanding (76%) and confidence (68%) in policy processes. Similarly, public health practitioners in a UK study^41^ reported feeling inexperienced and ill-equipped for policy engagement, citing formal education preparation as a limiting factor. In this study, several participants defaulted to discussing behaviour change theories when asked about policy theories, further suggesting the need to strengthen the presence of policy process content in contemporary tertiary health promotion curricula.^42^ This supports broader calls to deepen competencies in applying theories of the policy process to guide healthy public policy action.^39^ Research examining how policy-related theory is currently embedded and taught in tertiary programs would be valuable in guiding curriculum development.

 Workforce capacity, staff turnover and limited resourcing were also identified by participants as challenges to effective policy engagement. These issues were complicated by limited external recognition of the health promotion profession more broadly. Practitioners reflected on long-standing concerns that the absence of formal industry accreditation undermines the perceived legitimacy and specialised skills of health promotion practitioners.^43^ While formal accreditation has recently been introduced in Australia, participants noted that it is not being widely recognised nor actively used in recruitment processes. Nonetheless, many valued accreditation for supporting continuous professional development and maintaining contemporary practice standards. Given that existing global competency frameworks also emphasise policy development and assessment as core skills,^11-13^ formal accreditation may further reinforce the importance of policy competencies and provide impetus for ongoing professional development in policy process theories.^44^ Evidence suggesting a positive correlation between knowledge of public policy processes and engagement in policy action^45^ underscores the need for capacity building initiatives.^22^ Future practice and research may benefit from further exploration of the design and impact of continuing professional development initiatives and accreditation mechanisms in reinforcing policy competencies among health promotion practitioners.^46,47^ A review of how policy competencies are articulated within existing frameworks may also be warranted to ensure alignment with the evolving demands of contemporary health promotion practice.

## Strengths and limitations

 A key strength of the study was the inclusion of health promotion practitioners with varied experience levels, enabling rich exploration of perspectives across the career spectrum from early career to those with extensive tenure. The qualitative methodology also facilitated deep insight into health promotion practitioner experiences. However, limitations include the small purposive sample drawn largely from the researchers’ professional networks, which may not represent the broader workforce. The predominance of highly experienced practitioners (median of 18 years) may have biased findings toward senior viewpoints. Pre-existing professional relationships between researchers and participants may have influenced both participation and disclosure. Broader sampling across jurisdictions and sectors could strengthen future research. Additionally, none of the researchers had formal political science training, which may have influenced positionality and interpretation of data, particularly regarding policy processes theory nuances.

## Conclusion

 Limited evidence exists globally regarding health promotion practitioners’ engagement with policy processes, despite healthy public policymaking being central to the profession. This study, based in one jurisdiction in Australia, identified gaps in practitioners’ capacity to apply policy theory in practice, consistent with the limited international evidence. Opportunities exist to bridge the gap between theory and practice through stronger inclusion of theories of the policy process within tertiary education, formalising industry accreditation, and strengthening professional development systems. These strategies can support health promotion practitioners in Australia and internationally to enhance and maintain the skills required to meet industry core competencies needed for effective policy engagement. There is a need, nationally and internationally, to deepen our understanding of health promotion policy practice and identify the actions required across all levels and settings to better support practitioners’ engagement in policy processes. Strengthening the systems that enable health promotion policy action is essential to advancing healthy public policy and addressing the complex determinants of health. Future efforts could include developing tailored professional development programs, examining how theoretical knowledge of policy processes can be operationalised in practice, and exploring how organisational, workforce, and system-level supports can contribute to building a skilled, adaptable, and policy-literate global health promotion workforce.

## Competing Interests

 The authors declare that they have no conflict of interest.

## Ethical Approval

 Ethical approval was granted by the University of the Sunshine Coast Human Research Ethics Committee (Reference number: S231868). A full and detailed explanation of the study was provided, and all participants provided written informed consent prior to commencing participation. This study complied with the basic ethical principles contained in the Helsinki Declaration and its amendments.^48^

## Supplementary Files

Supplementary file 1. Semi-structured interview guide
