# Supplementary material for: Exploring Australian Health Promotion Practitioners’ Awareness, Understanding and Application of Theories of the Policy Process in Health Promotion Practice: A Qualitative Study
Source: Health Promot Perspect. 2026 Jun 6;16(1):101–10. doi: 10.34172/hpp.45504 (PMC13402642; doi:10.34172/hpp.45504)
Supplement: Supplementary file 1 — Semi-structured interview guide [file hpp-16-101-s001.pdf]

Jones et al, Health Promotion Perspectives 2026;16(1):101-S1
doi:10.34172/hpp.45504
https://hpp.tbzmed.ac.ir

In-depth, semi-structured interview guide

Introductions between interviewer and interviewee (if not already known)

Summarise the purpose of the study

Give interviewee opportunity to ask any questions about the research

Set voice-recorder/software to record interview

These first questions that you will be asked will provide an overview of your experience as a health promotion professional and your overall perspective and experiences of policy processes in health promotion.

Section 1 - Warm up questions

1. Please describe your experience as a health promotion professional and the health promotion activities that you have undertaken as part of your work.

2. What involvement have you had in policy processes to shape public health outcomes?

    a. *Possible follow up questions:*
        i. *Can you give me some examples?*
        ii. *What was the policy context – topic; type of policy; policy owner? How were you involved? How were others involved?*

3. Thinking about any formal education that you have undertaken in health promotion, on completing the qualification, how prepared did you feel in navigating policy processes?

    a. *Possible follow up question:*
        i. *How has this level of confidence changed since you have been working in health promotion?*
        ii. *What has influenced that change?*

The following questions are designed to explore your awareness of, understanding of, and practical application of policy process theories in your work as a health promotion professional.

Section 2 – Policy process theory awareness and understanding

4. In your experience, what factors influence policy decisions in health promotion practice?

5. Can you describe any policy process theories or frameworks that can be applied to health promotion practices?
    a. *Prompt: Have you heard of Multiple Streams Theory, Advocacy Coalition Framework, Punctuated Equilibrium Framework?*

       (If no – go to Q7)

<u>Section 3 – Policy process theory application</u>

6. Can you describe an example of how you have applied a specific policy process theory in your health promotion work? If yes, please specify the theory or theories.

    a. (IF answered Question 6 with a specific example) *How was the use of policy process theory useful for guiding health promotion actions in your work?*

7. In your opinion, what are some potential challenges or barriers to navigating policy processes in real-world health promotion initiatives?

    Possible follow-up questions:

    a. *Can you provide any specific examples or scenarios that illustrate these challenges?*
    b. *How do these challenges impact your ability to effectively engage in public policy processes?*
    c. *Are there any strategies or solutions you have found helpful in overcoming these challenges?*

This last question aims to explore your thoughts on how health promotion professionals can be further supported to engage with policy process theories in their work.

<u>Section 4 – closing questions</u>

8. What additional resources or support do you think may be useful for health promotion practitioners to effectively engage in healthy public policy processes?

    Possible follow-up questions:

    a. *Can you provide any specific examples of the types of resources or support you believe would be beneficial?*
    b. *In what ways do you think these additional resources or support would enhance your ability to engage in healthy public policy processes?*
    c. *Are there any existing initiatives or programs in other areas that you believe could serve as models for providing the necessary support?*

The final questions are to get a bit of information to provide context for our study.

9. What qualifications have you completed in health promotion?

10. How many years of experience do you have working in health promotion?

11. Is there anything else that you would like to add that I haven't asked about?
